# High AKAP8L expression predicts poor prognosis in esophageal squamous cell carcinoma

**DOI:** 10.1186/s12935-022-02492-3

**Published:** 2022-02-21

**Authors:** Qiu-yun Luo, Tian Di, Miao-Zhen Qiu, Zeng-fei Xia, Yong Du, Run-duan Lin, Li-qiong Yang, Yu-ting Sun, Da-Jun Yang, Jian Sun, Lin Zhang

**Affiliations:** 1 State Key Laboratory of Oncology in South China, Collaborative Innovation Center for Cancer Medicine, Sun Yat-Sen University Cancer Center, 651 Dongfeng Road East, Guangzhou, 510060 P. R. China; 2 Department of Experimental Research, Sun Yat-Sen University Cancer Center, 651 Dongfeng Road East, Guangzhou, 510060 P. R. China; 3 The Eighth Affiliated Hospital, Sun Yat-Sen University, Shenzhen, 518033 China; 4 Department of Medical Oncology, Sun Yat-Sen University Cancer Center, Guangzhou, 510060 China; 5 Department of General Affairs Office, Sun Yat-Sen University Cancer Center, Guangzhou, 510060 China; 6 Department of Clinical Laboratory, Sun Yat-Sen University Cancer Center, Guangzhou, 510060 China; 7 Department of Clinical Research, The Third Affiliated Hospital, Sun Yat-Sen University, Guangzhou, 510060 China

**Keywords:** AKAP8L, Esophageal squamous cell carcinoma, Prognosis biomarker, Bioinformatic analysis

## Abstract

**Background:**

Esophageal squamous cell carcinoma (ESCC) is a severe disease with high mortality, and is associated with poor prognosis and frequent lymphatic metastasis. Therefore, prognostic indicators for ESCC are urgently needed. A-kinase anchor-protein 8-like (AKAP8L) is a member of the A kinase anchor-protein (AKAPs) family and is overexpressed in many cancers. However, the role of AKAP8L in ESCC remains unclear. The aim of this study is to investigate the expression patterns and prognostic value of AKAP8L in ESCC.

**Methods:**

The mRNA expression of AKAP8L was analyzed from the dataset of The Cancer Genome Atlas (TCGA) and Gene Expression Omnibus (GEO). Immunohistochemistry was applied to detect the AKAP8L expression in tissue microarray. Pearson’s chi-square test was carried out for the correlation analysis of clinicopathological features and AKAP8L expression. The prognostic significance of clinicopathological features and AKAP8L expression was determined by univariate and multivariate Cox hazard models. Kaplan–Meier survival curve was used for survival analysis.

**Results:**

We found that the mRNA level of AKAP8L was higher in tumor tissues than in adjacent tissues in TCGA and GEO dataset. High AKAP8L expression was associated with poor overall survival (OS) in ESCC patients (*p* = 0.0039). Besides, AKAP8L expression was highly expressed in patients with lymph node metastasis detected by ESCC tissue microarray (*p* = 0.0014). The comparison of the different clinicopathological features of ESCC between high and low AKAP8L expression groups revealed that high AKAP8L expression was related to lymph node stage (*p* = 0.041). Kaplan–Meier survival analysis revealed that high AKAP8L expression indicates an unfavorable progression-free survival (PFS) and OS in ESCC patients (*p* < 0.0001). Univariate and multivariate analyses confirmed that AKAP8L was an independent prognostic factor for PFS and OS in ESCC (*p* = 0.003 and *p* < 0.0001).

**Conclusions:**

In conclusion, this study demonstrated that high expression of AKAP8L is associated with poor prognosis of ESCC and can be considered an independent risk factor for ESCC.

## Introduction

Esophageal cancer is one of the commonest cancers and is associated with a high mortality, poor prognosis, and frequent lymphatic metastasis [1, 2]. The survival rate is inversely correlated with metastasis and recurrence in esophageal cancer [3]. The two subtypes of esophageal carcinoma, based on histopathological characteristics, are esophageal squamous cell carcinoma (ESCC) and esophageal adenocarcinoma (EAC) [4]. In China, ESCC constitutes the most important pathological type and causes for over 90% of all esophageal cancer cases [5]. Despite the improved 5-year survival rate of ESCC patients in the past decade, the prognosis of ESCC remains poor because most patients are diagnosed at an advanced stage [6, 7]. Therefore, the identification of novel biomarkers for early diagnosis and better prognosis of ESCC is urgently needed.

A-kinase anchoring proteins (AKAPs) belong to a family of structurally diverse scaffold proteins that share a common function of binding the cAMP-dependent protein kinase A (PKA) and anchoring the regulatory (R) subunit of PKA near the substrate [8, 9]. Moreover, many AKAPs interact with other proteins, such as other scaffold proteins, phosphatases, ion channels, and receptors. Recent research has increasingly focused on members of the AKAP family and their functions. AKAP8 is a core member of the AKAP family and has multiple activities [10] that include integrated regulation of transcription and RNA splicing through the binding of its zinc finger domain (ZF) to many factors during RNA processing and transcription [11, 12]. Recent study has found that AKAP8 could inhibit tumor metastasis by regulating the splice isoform switching of CLSTN1, a key molecule that affects epithelial–mesenchymal transition [13]. However, another research team revealed that AKAP8 may promote tumorigenesis through the formation of phase-separated and liquid-like condensates in the cancer cell nucleus and regulating alternative splicing [14].

The protein sequence homology of AKAP8L and AKAP8 is as high as 61%, which suggests the possibility that AKAP8L has similar functions with AKAP8 in tumorigenesis and metastasis. A previous study revealed that AKAP8L interacts with mTORC1 and facilitates mTORC1-promoted cell growth [15]. However, no study has investigated the expression profile of AKAP8L in human cancer, and the function and clinical significance of AKAP8L in ESCC remain unclear.

Therefore, in this study, we first comparatively analyzed the mRNA expression level of AKAP8L in ESCC and non-tumoral tissues. Next, the AKAP8L expression profile in an ESCC tissue microarray was detected by immunohistochemistry, and the correlation between AKAP8L expression levels and the clinical characteristics and survival outcome of ESCC patients was studied to clarify the clinical significance of AKAP8L in ESCC patients.

## Materials and methods

### AKAP8L gene expression analysis

The mRNA expression of AKAP8L in ESCC and non-tumoral tissues was ascertained from The Cancer Genome Atlas (TCGA) as well as from two Gene Expression Omnibus (GEO) data sets. The RNA-seq data was downloaded from TCGA database (https://portal.gdc.cancer.gov/). GSE23400 and GSE20347 contained gene expression data of 53 and 17 pairs of tumor-adjacent normal specimens and tumor biopsy specimens from ESCC patients that were downloaded from the GEO database (http://ncbi.nlm.nih. gov/geo/). The mRNA expression level of AKAP8L was comparatively analyzed in the normal and tumoral tissues.

### Kaplan–Meier plot analysis

Kaplan–Meier plot (http://kmplot.com/analysis/) was applied to analyze the relationship between AKAP8L gene expression and survival rates in ESCC (n = 81) based on the hazard ratios (HR) and log-rank *P*-values.

### Tissue microarray

A tissue microarray containing 116 human ESCC sample tissues that were collected between May 2000 and December 2002 at Sun Yat-sen University Cancer Center in China was obtained. The study was approved by the Ethics Committee of The Sun Yat-sen University Cancer Center. All participants provided written informed consent for study participation. The clinical characteristics of patients are listed in Table 1.

**Table 1:** Clinical data of 116 cases of esophageal squamous cell carcinoma

Characteristics	Total patients (n, %)
Gender	
Female	20 (17.2%)
Male	96 (82.8%)
Age, year	
<60	70 (60.3%)
≥60	46 (39.7%)
Differentiation grade	
I	34 (29.3%)
II	52 (44.8%)
III	30 (25.9%)
T stage	
T1	4 (3.4%)
T2	30 (25.7%)
T3	78 (67.2%)
T4	4 (3.4%)
N stage	
N0	58 (50.0%)
N1	57 (49.1%)
N2	1 (0.9%)
M stage	
M0	110 (94.8%)
M1	6 (5.2%)
Clinical stage	
I	3 (2.6%)
II	61 (52.6%)
III	46 (39.6%)
IV	6 (5.2%)

### Immunohistochemistry

Paraffin-embedded tissue sections were dewaxed in dimethyl benzene and rehydrated in gradient ethanol solutions. Thereafter, 3% H_2_O_2_ was applied for 10 min to inhibit the endogenous peroxidase, and the antigens were retrieved by microwave heating. After cooled to room temperature, the tissue sections were blocked and incubated with 10% fetal bovine serum for 30 min. The primary antibody AKAP8L (1:200; HPA042485) was added and incubated at 4 °C overnight, and the tissue sections were subsequently incubated with HRP-labeled universal anti-mouse/rabbit secondary antibody in the dark. Immunostaining was visualized with diaminobenzidine (DAB), and the tissue sections were counterstained with hematoxylin, then dehydrated and mounted.

### Immunohistochemical scoring

Based on the proportions of positive cells and staining intensity, the level of AKAP8L expression was assessed by two independent pathologists who were blinded to the clinicopathological information. The proportion of positive cells was graded as 0 (0%–5%), 1 (6%–25%), 2 (26%–50%), 3 (51%–75%), and 4 (76%–100%), whereas staining intensity was rated as 1 (weak), 2 (moderate), and 3 (strong). The IHC scores of AKAP8L expression levels were based on the multiplication of proportions of positive cells and staining intensity. The final AKAP8L expression was defined as follows: negative (−), 0 points; low expression ( +), 1–4 points; moderate (+ +), 5–8 points; and high expression (+ + +), 9–12 points. For further investigation, all ESCC patients were divided into two groups of low and high AKAP8L expression according to an AKAP8L median IHC score of 4.

### Statistical analysis

GraphPad Prism (GraphPad Software, Inc.) and SPSS (IBM, USA) were used for data analysis. The Student’s *t*-test was used for intergroup comparisons. The Mann–Whitney *U* test was used to compare nonnormally distributed data. The area under the curve (AUC) value was computed based on receiver operating characteristic (ROC) analysis using the pROC package in R version 3.6.1. Pearson’s chi-square test was used for the correlation analysis of clinicopathological features and AKAP8L expression. Kaplan–Meier survival curve was used for survival analysis. Univariate and multivariate analyses were carried out based on Cox proportional hazard regression. *P*-value < 0.05 was considered statistically significant.

## Results

### mRNA expression level of AKAP8L in ESCC

**Fig. 1:**
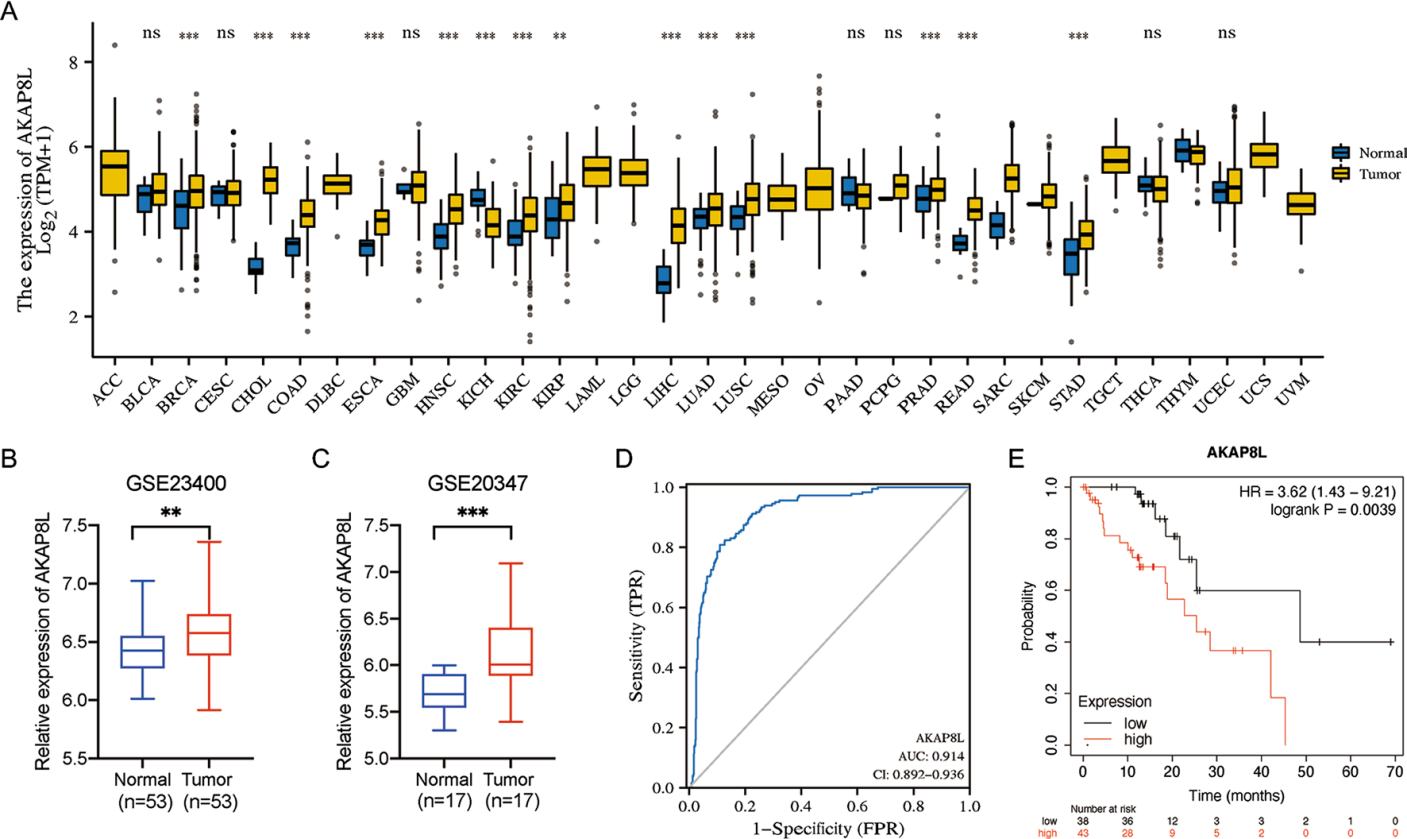
The expression profile of AKAP8L in esophageal squamous cell carcinoma. **A** High or low expression of AKAP8L in different human cancer tissues compared with normal tissues from the TCGA database. **B**, **C** The expression level of AKAP8L was higher in ESCC tissue than the adjacent normal tissue in GSE23400 dataset and GSE20347 dataset. **D** ROC curve indicated that AKAP8L showed high accuracy in predicting normal and tumor outcomes. E. High AKAP8L expression was correlated with poor OS in ESCC using the Kaplan–Meier plotter database (n = 81). *P < 0.05, **P < 0.01, ***P < 0.001

We first analyzed the gene expression levels of AKAP8L in different human cancer tissues as compared with those in normal tissues based on data obtained from the TCGA database. The mRNA expression of AKAP8L was significantly higher in multiple type of cancer tissues than in the corresponding normal tissues, including esophageal carcinoma (ESCA) (Fig. 1A). The level of AKAP8L expression was further validated in two independent GEO datasets: GSE23400 and GSE20347. AKAP8L expression was higher in ESCC tumor tissues than in normal tissues in both datasets (Fig. 1B and C). ROC analysis revealed that AKAP8L possessed high sensitivity and specificity for predicting patient outcomes (AUC 0.914; Fig. 1D). In addition, the prognostic value of AKAP8L expression was analyzed in ESCC using the Kaplan–Meier plotter database. High AKAP8L expression correlated with poorer prognosis in ESCC (hazard ratio [HR] = 3.62, 95% confidence interval [CI] = 1.43 to 9.21, *p* = 0.0039; Fig. 1E).

### AKAP8L expression levels in tissue samples of ESCC patients

**Fig. 2:**
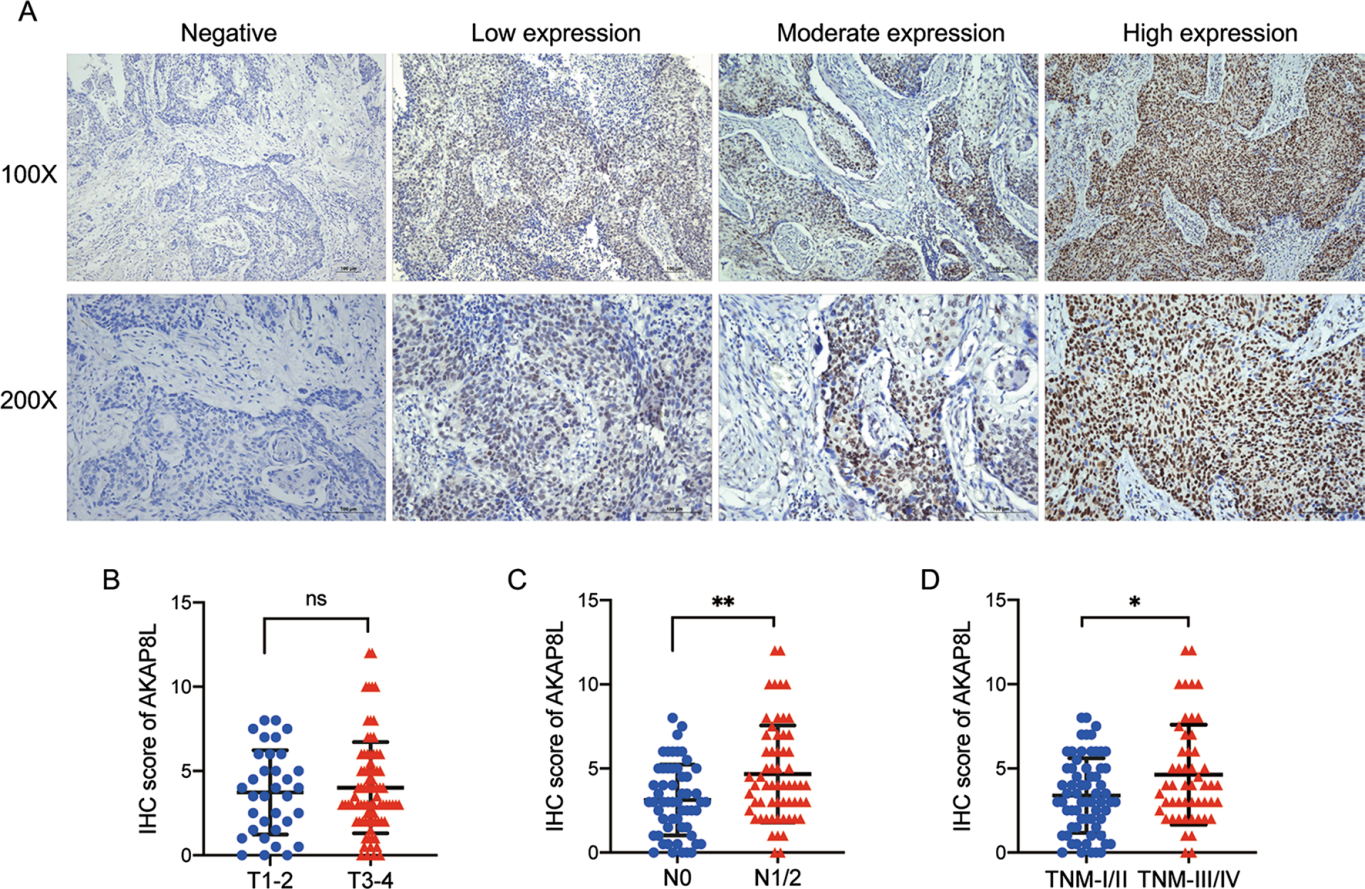
Representative images and statistical analysis of immunohistochemical staining for AKAP8L in ESCC tissues. **A** Representative images of IHC staining of AKAP8L with negative, weak, moderate, and strong expression. Scale bar: 100 μm. **B** Statistical analysis of AKAP8L expression in T1/2 and T3/4 stage ESCC samples based on IHC scores. **C** The expression level of AKAP8L in patients with lymph node metastasis was significantly higher than that in patients without lymph node metastasis. **D** The expression level of AKAP8L in TNM stage III/IV patients was significantly higher than that in TNM stage I/II patients

Next, we evaluated the level of AKAP8L expression in the ESCC tissue microarray by immunohistochemical assay. The results showed that positive staining of AKAP8L was predominately present in the nucleus of tumor cells, which was observed in 93.97% (109/116) of ESCC tissues. Figure 2A shows different IHC staining intensities of AKAP8L. The AKAP8L IHC scores were comparable between ESCC patients in the T1–2 and T3–4 stages (Fig. 2B). The IHC score of AKAP8L was significantly higher in N1/2 ESCC than in N0 ESCC patients (Fig. 2C). Furthermore, TNM stage III/IV ESCC patients had higher AKAP8L expression than those with TNM I/II ESCC (Fig. 2D).

### Correlation between AKAP8L expression and clinical characteristics of ESCC patients

We further analyzed the correlations between the AKAP8L expression levels and the clinicopathological parameters of the 116 ESCC patients. The median IHC score of 4 was applied as the cut-off value for AKAP8L expression. AKAP8L expression was high in 55 (47.41%) tissue samples of ESCC patients. We next evaluated the association of AKAP8L expression in tumor tissues with the following clinicopathologic parameters: gender, the age of diagnosed, tumor-differentiation grade, T stage, N stage, distant metastasis, and clinical stage. The correlation between clinicopathological features and AKAP8L expression in 116 ESCC patients are summarized in Table 2. High AKAP8L expression was associated with a higher N stage than was low AKAP8L expression (*p* = 0.041). AKAP8L expression level showed no significant correlation with other clinicopathologic features.

**Table 2:** Correlation between the expression of AKAP8L and clinicopathologic parameters in esophageal squamous cell carcinoma

Characteristics	Total	AKAP8L expression		*p* value
		Low (n = 61)	High (n = 55)	
Gender				
Female	20	10	10	0.799
Male	96	51	45	
Age at diagnosis				
< 60	70	39	31	0.405
≥ 60	46	22	24	
Differentiation grade				
I–II	86	44	42	0.603
III	30	17	13	
Tumor extent				
T1/T2	34	17	17	0.719
T3/T4	82	44	38	
Lymph node stage				
N0	58	36	22	0.041
N1/2	58	25	33	
Distant metastasis				
M0	110	60	50	0.081
M1	6	1	5	
Clinical stage				
I/II	64	38	26	0.104
III/IV	52	23	29	

### Prognostic value of AKAP8L in ESCC

In order to determine whether AKAP8L expression and the clinicopathological characteristics can served as independent risk factors for ESCC patients, univariate and multivariate analyses were performed based on Cox regression analysis. The univariate analysis indicated that age < 60 years (*p* = 0.001), T3/4 stage (*p* = 0.044), N1/2 stage (*p* < 0.001), TNM III/IV stage (*p* < 0.001), and high AKAP8L expression level (*p* < 0.001) were significantly associated with a decrease in progression-free survival (PFS; Table 3). Multivariate Cox model analysis showed that age (*p* = 0.009), N stage (*p* = 0.005), and AKAP8L levels (*p* = 0.003) were independent predictors for PFS. Univariate analysis showed that age < 60 years (*p* = 0.003), N1/2 stage (*p* < 0.001), TNM III/IV stage (*p* = 0.001), and high AKAP8L expression level (*p* < 0.0001) were significantly associated with decreased overall survival (OS; Table 4). Moreover, multivariate Cox analysis indicated that age (*p* = 0.018), N stage (*p* = 0.011), and the AKAP8L levels (*p* < 0.0001) were independent predictors of OS.

**Table 3:** Univariate and multivariate Cox regression analysis of progression-free survival in patients with esophageal squamous cell carcinoma

Variable	Univariate analysis		Multivariate analysis	
	HR (95% CI)	*p* value	HR (95% CI)	*p* value
Gender (Female vs. Male)	1.148 (0.680–1.938)	0.606		
Age (< 60 vs. ≥ 60)	1.936 (1.289–2.908)	***0.001***	1.737 (1.145–2.633)	***0.009***
Differentiation grade (III vs. I–II)	0.851 (0.539–1.344)	0.490		
T stage (T3–4 vs. T1–2)	1.593 (1.013–2.504)	***0.044***	1.634 (0.920–2.903)	0.094
N stage (N1–2 vs. N0)	2.709 (1.788–4.106)	** < *0.0001***	2.707 (1.355–5.407)	***0.005***
M stage (M1 vs. M0)	1.251 (0.545–2.872)	0.597		
Clinical stage (III–IV vs. I–II)	2.122 (1.413–3.189)	** < *0.0001***	0.702 (0.324–1.518)	0.368
AKAP8L expression (High vs. Low)	2.192 (1.449–3.317)	** < *0.0001***	1.943 (1.258–3.000)	***0.003***

**Table 4:** Univariate and multivariate Cox regression analysis of overall survival in patients with esophageal squamous cell carcinoma

Variable	Univariate analysis		Multivariate analysis	
	HR (95% CI)	*p* value	HR (95% CI)	*p* value
Gender (Female vs. Male)	1.127 (0.665–1.911)	0.656		
Age (< 60 vs. ≥ 60)	1.887 (1.242–2.866)	***0.003***	1.682 (1.095–2.586)	***0.018***
Differentiation grade (III vs. I–II)	0.789 (0.497–1.253)	0.315		
T stage (T3–4 vs. T1–2)	1.461 (0.920–2.320)	0.108		
N stage (N1–2 vs. N0)	2.598 (1.695–3.982)	** < *0.0001***	2.252 (1.206–4.208)	***0.011***
M stage (M1 vs. M0)	1.250 (0.544–2.873)	0.599		
Clinical stage (III–IV vs. I–II)	1.980 (1.304–3.006)	***0.001***	0.956 (0.524–1.744)	0.883
AKAP8L expression (High vs. Low)	2.588 (1.677–3.992)	** < *0.0001***	2.208 (1.417–3.440)	** < *0.0001***

### Association between AKAP8L expression and survival outcome in ESCC

**Fig. 3:**
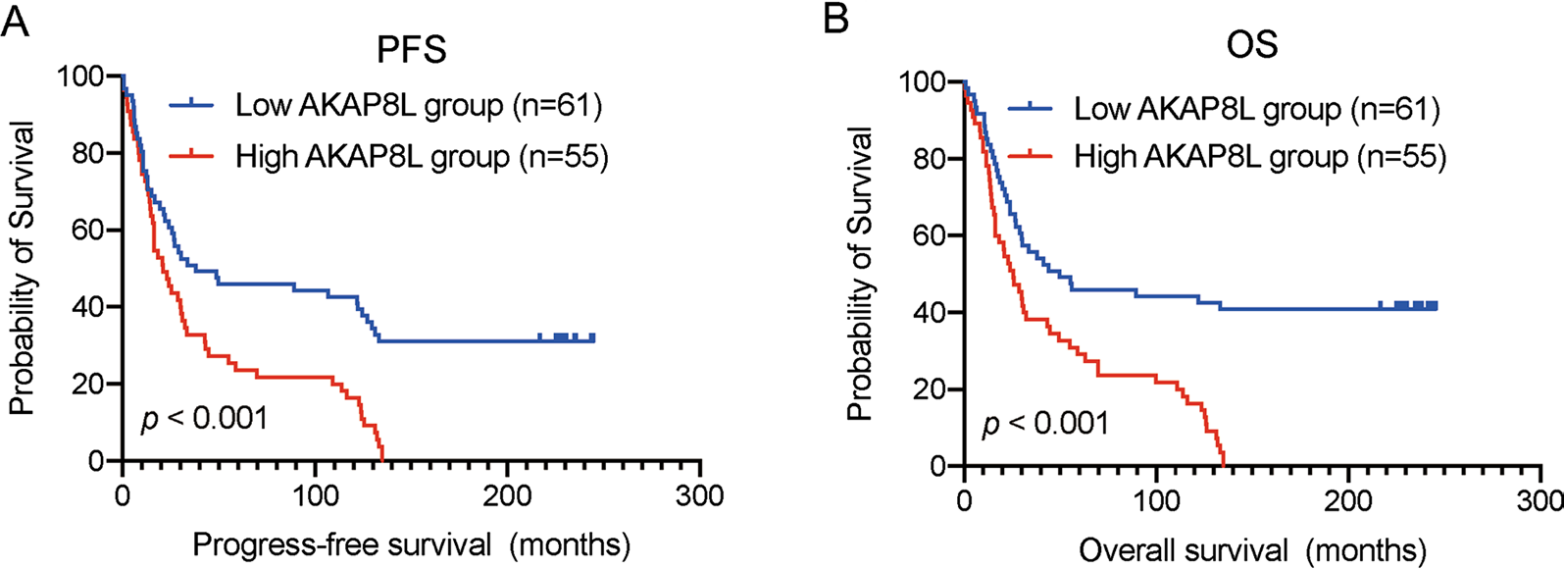
Kaplan–Meier survival curves grouped by high and low AKAP8L expression in ESCC patients. **A** Progression-Free-Survival (PFS) in all patients. **B** Overall survival (OS) in all patients

**Fig. 4:**
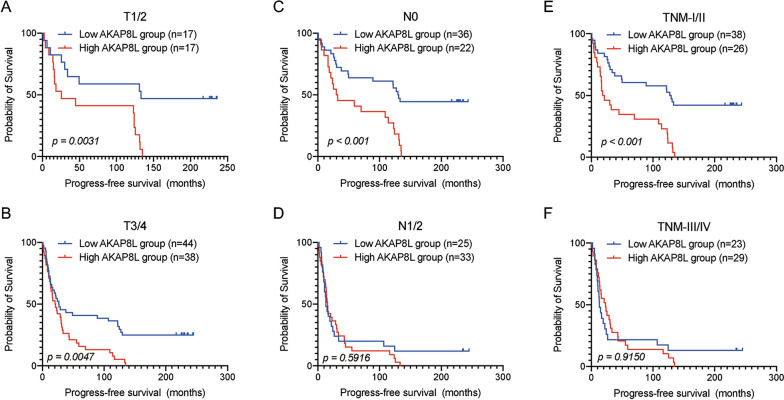
Kaplan–Meier survival curves for progress-free survival in ESCC patients with high and low AKAP8L expression stratified by different clinical factors. Kaplan–Meier survival for PFS in subgroups stratified by T1/2 (**A**), T3/4 (**B**), N0 (**C**), N1/2 (**D**), TNM stage I/II (**E**) and stage TNM stage III/IV (**F**) in ESCC patients

**Fig. 5:**
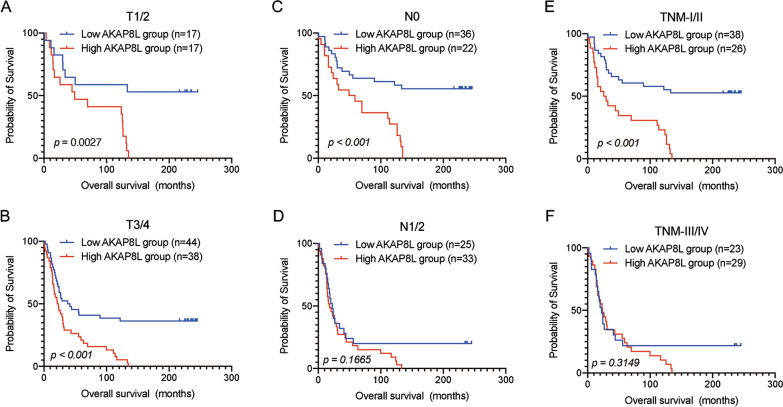
Kaplan–Meier survival curves for overall survival in ESCC patients with high and low AKAP8L expression stratified by different clinical factors. Kaplan–Meier survival for OS in subgroups stratified by T1/2 (**A**), T3/4 (**B**), N0 (**C**), N1/2 (**D**), TNM stage I/II (**E**) and stage TNM stage III/IV (**F**) in ESCC patients

We assessed the prognostic performance of AKAP8L in predicting the PFS and OS outcome of all ESCC cases. Kaplan–Meier survival analysis showed that patients in the low AKAP8L expression group had better survival results with regard to PFS and OS (Fig. 3). Next, we analyzed the PFS outcome between the low and high AKAP8L expression groups in patients with different phenotypes (Fig. 4). High AKAP8L expression predicted poorer PFS outcome in ESCC patients with T1/2 stage (*p* = 0.0031), T3/4 stage (*p* = 0.0047), N0 (*p* < 0.001), and TNM I /II ((*p* < 0.001), whereas the PFS survival outcome did not differ significantly between patients in the low and high AKAP8L-expression groups in N1/2 (*p* = 0.5916) and TNM III /IV (*p* = 0.9150). Furthermore, the OS outcome analysis in Fig. 5 revealed that high AKAP8L expression correlated with poorer survival results in patients with T1/2 stage (*p* = 0.0027), T3/4 stage (*p* < 0.001), N0 (*p* < 0.001), and TNM I/II stage (*p* < 0.001), but not with the N1/2 stage (*p* = 0.1665) and TNM III/IV stage (*p* = 0.3149).

## Discussion

ESCC is a common malignancy in China, and accounts for nearly half of all new ESCC cases and deaths worldwide [16]. Despite considerable progress in surgical resection, radiotherapy, chemotherapy, and immunotherapy for ESCC, the clinical efficacy of these therapeutic options for locally advanced ESCC remain disappointing [17, 18]. Thus, there is an urgent need to explore effective biomarkers for better prognostic prediction for ESCC patients.

Recently, an increasing number of studies have suggested that the signal-transduction complexes which regulated by AKAP family proteins are associated with cancer development and progression, thus, targeting AKAP may be a therapeutic option [19, 20]. For instance, evaluated AKAP4 expression is found in multiple tumors, and high AKAP4 expression positively impacts cell proliferation, migration, and invasion abilities in several cancers, including cervical cancer, colorectal cancer, and ESCC [21–24]. In addition, AKAP8 is upregulated in many types of cancer, including ovarian, lung, and esophageal squamous carcinoma, and mediates cell-cycle regulation and mitotic DNA condensation [25–27]. AKAP9 and AKAP13 regulate cell proliferation and epithelial–mesenchymal transition [28–31]. Therefore, the study of AKAP family proteins is helpful to enrich our understanding of the molecular mechanism of tumorigenesis, development, and prevention.

AKAP8L is a nuclear protein that was originally identified as a 95 kDa apparent molecular weight homolog similar to AKAP95, and thus was also named HA95 (homolog of AKAP95) [32]. The amino acid sequence homology of AKAP8L and AKAP95 was 61%. AKAP8 is involved in regulating the histone H3 lysine 4 (H3K4) histone methyltransferase (HMT) complexes through interacting with the WDR5 protein and DPY30 protein, which are core subunit of H3K4 HMT complexes [33]. Similar to AKAP8, AKAP8L also associates with the core subunits of H3K4 HMT complexes and may be a potential regulator of H3K4 methylation [33]. However, AKAP8L does not interact with DPY30 protein directly, further study is needed to identify the subunits of HMT complexes interacting with AKAP8L. AKAP95 and AKAP8L could be recruited to mitotic chromosomes and involved in mitotic progression, but the mechanism remains obscure [32, 34, 35]. Yun Li and their research team found that AKAP8L can interacted with AKAP95 and histone deacetylase 3 (HDAC3), and this interaction is increased during mitosis [25]. Depletion of AKAP8/AKAP8L or HDAC3 caused a similar impairment of mitotic progression and induced G2/M arrest. This provides new insights into the mechanism by which HDAC inhibitors are used to target HDAC3 in cancer treatment [25]. Another study found that knockdown of AKAP8L resulted in delayed maturation of erythrocyte progenitor cells, suggesting that AKAP8L may play a key role in regulating terminal differentiation of human primary erythrocytes, especially in regulating pyknosis and enucleation of erythroblasts. Further studies of AKAP8L in the future may help to provide a comprehensive understanding of the underlying mechanisms of nuclear rupture and the diversity of cell stages in different types of anemia [36]. AKAP8L is involved in many biological processes, but the function of AKAP8L in tumor development is unclear. Therefore, investigating AKAP8L expression profile in tumor tissues and its impact on clinical prognosis may help us understand its functional role.

In this study, we initially analyzed the mRNA expression level of AKAP8L in a pan-cancer sample obtained from the TCGA dataset. We found that, compared with normal tissues, AKAP8L mRNA levels increased significantly in many cancers, including ESCC. The differential expression of AKAP8L in ESCC was further verified in two independent GEO datasets. In addition, TCGA data showed that a higher level of AKAP8L expression was associated with a lower survival rate in ESCC. IHC staining of AKAP8L in a tumor tissue microarray of ESCC patients revealed higher AKAP8L expression in ESCC patients with lymphatic metastasis. High AKAP8L expression levels significantly correlated with N categories, but not with sex, age, histological differentiation grade, and tumor status or TNM stage of ESCC patients. Importantly, ESCC patients with high AKAP8L expression exhibited significantly shorter OS and PFS duration than those with low AKAP8L expression. These results indicate that AKAP8L may play an important role in tumor metastasis and progression of ESCC. However, the biological function of AKAP8L in promoting ESCC development and the underlying regulatory mechanisms warrant further investigation.

In conclusion, our study demonstrated that AKAP8L is highly expressed in ESCC tissues and high AKAP8L expression significantly correlates with lymphatic metastasis and poor survival outcome in ESCC. Therefore, AKAP8 may be a useful indicator of the prognosis of ESCC patients.

## Data Availability

The key raw data have been deposited into the Research Data Deposit (http://www.researchdata.org.cn), with the Approval Number of RDDB2022695799 and the datasets used in this study are publicly available.

## References

[1] Bray F, Ferlay J, Soerjomataram I, Siegel RL, Torre LA, Jemal A (2018). Global cancer statistics 2018: GLOBOCAN estimates of incidence and mortality worldwide for 36 cancers in 185 countries. CA Cancer J Clin.

[2] Malhotra GK, Yanala U, Ravipati A, Follet M, Vijayakumar M, Are C (2017). Global trends in esophageal cancer. J Surg Oncol.

[3] Peyre CG, Hagen JA, DeMeester SR, Altorki NK, Ancona E, Griffin SM, Holscher A, Lerut T, Law S, Rice TW (2008). The number of lymph nodes removed predicts survival in esophageal cancer: an international study on the impact of extent of surgical resection. Ann Surg.

[4] Huang FL, Yu SJ (2018). Esophageal cancer: Risk factors, genetic association, and treatment. Asian J Surg.

[5] Chen W, Zheng R, Zhang S, Zeng H, Fan Y, Qiao Y, Zhou Q (2014). Esophageal cancer incidence and mortality in China, 2010. Thorac Cancer.

[6] Ohashi S, Miyamoto S, Kikuchi O, Goto T, Amanuma Y, Muto M (2015). Recent advances from basic and clinical studies of esophageal squamous cell carcinoma. Gastroenterology.

[7] Mariette C, Finzi L, Piessen G, Van Seuningen I, Triboulet JP (2005). Esophageal carcinoma: prognostic differences between squamous cell carcinoma and adenocarcinoma. World J Surg.

[8] Tasken K, Aandahl EM (2004). Localized effects of cAMP mediated by distinct routes of protein kinase A. Physiol Rev.

[9] Wong W, Scott JD (2004). AKAP signalling complexes: focal points in space and time. Nat Rev Mol Cell Biol.

[10] Eide T, Coghlan V, Orstavik S, Holsve C, Solberg R, Skalhegg BS, Lamb NJ, Langeberg L, Fernandez A, Scott JD (1998). Molecular cloning, chromosomal localization, and cell cycle-dependent subcellular distribution of the A-kinase anchoring protein, AKAP95. Exp Cell Res.

[11] Hu J, Khodadadi-Jamayran A, Mao M, Shah K, Yang Z, Nasim MT, Wang Z, Jiang H (2016). AKAP95 regulates splicing through scaffolding RNAs and RNA processing factors. Nat Commun.

[12] Jiang H, Lu X, Shimada M, Dou Y, Tang Z, Roeder RG (2013). Regulation of transcription by the MLL2 complex and MLL complex-associated AKAP95. Nat Struct Mol Biol.

[13] Hu X, Harvey SE, Zheng R, Lyu J, Grzeskowiak CL, Powell E, Piwnica-Worms H, Scott KL, Cheng C (2020). The RNA-binding protein AKAP8 suppresses tumor metastasis by antagonizing EMT-associated alternative splicing. Nat Commun.

[14] Li W, Hu J, Shi B, Palomba F, Digman MA, Gratton E, Jiang H (2020). Biophysical properties of AKAP95 protein condensates regulate splicing and tumorigenesis. Nat Cell Biol.

[15] Melick CH, Meng D, Jewell JL (2020). A-kinase anchoring protein 8L interacts with mTORC1 and promotes cell growth. J Biol Chem.

[16] Ferlay J, Colombet M, Soerjomataram I, Mathers C, Parkin DM, Pineros M, Znaor A, Bray F (2019). Estimating the global cancer incidence and mortality in 2018: GLOBOCAN sources and methods. Int J Cancer.

[17] Abnet CC, Arnold M, Wei WQ (2018). Epidemiology of esophageal squamous cell carcinoma. Gastroenterology.

[18] Lagergren J, Smyth E, Cunningham D, Lagergren P (2017). Oesophageal cancer. Lancet.

[19] Reggi E, Diviani D (2017). The role of A-kinase anchoring proteins in cancer development. Cell Signal.

[20] Bucko PJ, Scott JD (2021). Drugs that regulate local cell signaling: AKAP targeting as a therapeutic option. Annu Rev Pharmacol Toxicol.

[21] Jagadish N, Parashar D, Gupta N, Agarwal S, Purohit S, Kumar V, Sharma A, Fatima R, Topno AP, Shaha C (2015). A-kinase anchor protein 4 (AKAP4) a promising therapeutic target of colorectal cancer. J Exp Clin Canc Res.

[22] Li S, Qin X, Li Y, Guo A, Ma L, Jiao F, Chai S (2016). AKAP4 mediated tumor malignancy in esophageal cancer. Am J Transl Res.

[23] Saini S, Jagadish N, Gupta A, Bhatnagar A, Suri A (2013). A Novel Cancer Testis Antigen, A-Kinase Anchor Protein 4 (AKAP4) is a potential biomarker for breast cancer. PLos ONE.

[24] Saini S, Agarwal S, Sinha A, Verma A, Parashar D, Gupta N, Ansari AS, Lohiya NK, Jagadish N, Suri A (2013). Gene silencing of A-kinase anchor protein 4 inhibits cervical cancer growth in vitro and in vivo. Cancer Gene Ther.

[25] Li Y, Kao GD, Garcia BA, Shabanowitz J, Hunt DF, Qin J, Phelan C, Lazar MA (2006). A novel histone deacetylase pathway regulates mitosis by modulating Aurora B kinase activity. Genes Dev.

[26] Zhao SP, Yi M, Yuan YY, Zhuang WX, Zhang DC, Yu XY, Chen XX, Teng BG, Guan ZY, Zhang YX (2015). Expression of AKAP95, Cx43, CyclinE1 and CyclinD1 in esophageal cancer and their association with the clinical and pathological parameters. Int J Clin Exp Med.

[27] Qi F, Yuan Y, Zhi X, Huang Q, Chen Y, Zhuang W, Zhang D, Teng B, Kong X, Zhang Y (2015). Synergistic effects of AKAP95, Cyclin D1, Cyclin E1, and Cx43 in the development of rectal cancer. Int J Clin Exp Pathol.

[28] Diviani D, Raimondi F, Del Vescovo CD, Dreyer E, Reggi E, Osman H, Ruggieri L, Gonano C, Cavin S, Box CL (2016). Small-molecule protein-protein interaction inhibitor of oncogenic Rho signaling. Cell Chem Biol.

[29] Smith FD, Langeberg LK, Cellurale C, Pawson T, Morrison DK, Davis RJ, Scott JD (2010). AKAP-Lbc enhances cyclic AMP control of the ERK1/2 cascade. Nat Cell Biol.

[30] Hu ZY, Liu YP, Xie LY, Wang XY, Yang F, Chen SY, Li ZG (2016). AKAP-9 promotes colorectal cancer development by regulating Cdc42 interacting protein 4. Bba-Mol Basis Dis.

[31] Hurtado L, Caballero C, Gavilan MP, Cardenas J, Bornens M, Rios RM (2011). Disconnecting the Golgi ribbon from the centrosome prevents directional cell migration and ciliogenesis. J Cell Biol.

[32] Orstavik S, Eide T, Collas P, Han IO, Tasken K, Kieff E, Jahnsen T, Skalhegg BS (2000). Identification, cloning and characterization of a novel nuclear protein, HA95, homologous to A-kinase anchoring protein 95. Biol Cell.

[33] Bieluszewska A, Weglewska M, Bieluszewski T, Lesniewicz K, Poreba E (2018). PKA-binding domain of AKAP8 is essential for direct interaction with DPY30 protein. FEBS J.

[34] Steen RL, Cubizolles F, Le Guellec K, Collas P (2000). A kinase-anchoring protein (AKAP)95 recruits human chromosome-associated protein (hCAP)-D2/Eg7 for chromosome condensation in mitotic extract. J Cell Biol.

[35] Collas P, Le Guellec K, Tasken K (1999). The A-kinase-anchoring protein AKAP95 is a multivalent protein with a key role in chromatin condensation at mitosis. J Cell Biol.

[36] Huang P, Zhao Y, Zhong J, Zhang X, Liu Q, Qiu X, Chen S, Yan H, Hillyer C, Mohandas N (2020). Putative regulators for the continuum of erythroid differentiation revealed by single-cell transcriptome of human BM and UCB cells. Proc Natl Acad Sci USA.

